# Estimating and Modelling Bias of the Hierarchical Partitioning Public-Domain Software: Implications in Environmental Management and Conservation

**DOI:** 10.1371/journal.pone.0011698

**Published:** 2010-07-21

**Authors:** Pedro P. Olea, Patricia Mateo-Tomás, Ángel de Frutos

**Affiliations:** 1 School of Biology, IE University, Segovia, Spain; 2 Department of Biodiversity and Environmental Management, Faculty of Biological and Environmental Science, University of León, León, Spain; University of Liverpool, United Kingdom

## Abstract

**Background:**

Hierarchical partitioning (HP) is an analytical method of multiple regression that identifies the most likely causal factors while alleviating multicollinearity problems. Its use is increasing in ecology and conservation by its usefulness for complementing multiple regression analysis. A public-domain software “hier.part package” has been developed for running HP in R software. Its authors highlight a “minor rounding error” for hierarchies constructed from >9 variables, however potential bias by using this module has not yet been examined. Knowing this bias is pivotal because, for example, the ranking obtained in HP is being used as a criterion for establishing priorities of conservation.

**Methodology/Principal Findings:**

Using numerical simulations and two real examples, we assessed the robustness of this HP module in relation to the order the variables have in the analysis. Results indicated a considerable effect of the variable order on the amount of independent variance explained by predictors for models with >9 explanatory variables. For these models the nominal ranking of importance of the predictors changed with variable order, i.e. predictors declared important by its contribution in explaining the response variable frequently changed to be either most or less important with other variable orders. The probability of changing position of a variable was best explained by the difference in independent explanatory power between that variable and the previous one in the nominal ranking of importance. The lesser is this difference, the more likely is the change of position.

**Conclusions/Significance:**

HP should be applied with caution when more than 9 explanatory variables are used to know ranking of covariate importance. The explained variance is not a useful parameter to use in models with more than 9 independent variables. The inconsistency in the results obtained by HP should be considered in future studies as well as in those already published. Some recommendations to improve the analysis with this HP module are given.

## Introduction

**Figure 1:**
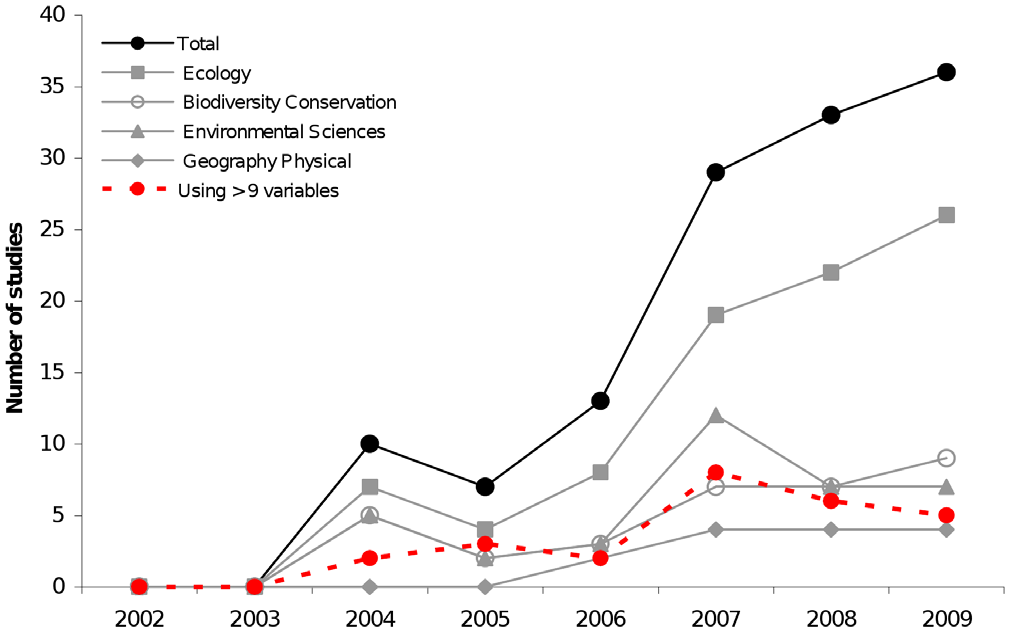
Number of studies using the HP package of Walsh and Mac Nally [**9**] for the R software over time. The subject categories with most number of studies are shown. Filled circles: number of total studies using HP package (*n* = 128). Filled squares: “Ecology” subject category. Opened circles: “Biodiversity conservation” subject category. Filled triangles: “Environmental sciences” subject category. Filled diamonds: “Geography physical” subject category. Red filled circles and dashed line: Number of studies using the HP package with more than 9 variables (*n* = 26). Note that a same study can pertain to more than 1 subject category and thus the sum of the number of studies from all the categories is higher than the number of total of studies.

In recent years, multiple regression analysis (e.g. Generalized Linear Models, GLMs) is widely used in ecology and conservation biology. However, this statistical approach to modelling can be seriously affected by multicollinearity between the explanatory variables, i.e. correlation among them [1]. Problems caused by collinearity have been traditionally reduced by removing highly correlated explanatory variables during model creation or by using principal components analysis (PCAs) taking the factors derived from the PCA as predictor variables [2]. Nonetheless, collinearity problems can be effectively alleviated using an analytical method named hierarchical partitioning (HP hereafter; [3]). HP reduces collinearity problems by determining the independent contribution of each explanatory variable to the response variable and separates it from the joint contribution, resulting from correlation with other variables (for a detailed explanation of how HP works, see [4], [5]).This allows ranking the importance of the covariates in explaining the response variable independently of the others covariates. Given its usefulness for complementing multiple regression analysis and by the recent developing of a free module (“hier.part package”) [6], [7] for running in R (a free statistical software) [8], the use of HP is increasing in different research fields. This increase can be measured using the Thomson Institute (ISI Web of Science) bibliographic database (1900–31 December 2009) to identify all papers indexed that cited Mac Nally and Walsh [4], [5], [9]. Using this bibliometric approach, we found a total of 128 studies that used this package distributed in 28 subject categories (Fig. 1; we had not access to 16 studies (5.4%) of 292 citing [4], [5], [9] for checking use of HP package). The increase in the number of papers was predominant in the “ecology” subject category and, to a lesser extent, in the “biodiversity conservation” and “environmental sciences” subject categories [2], [9]–[17] (Fig. 1).

HP produces, according to authors, a “minor rounding error” with models comprised of more than 9 explanatory variables [7], [18]. Several works have already used these HP modules for identifying important factors in biodiversity [2], [11], [19], conservation [10], [13], [15], environmental monitoring [20], forest management for conservation [21] and predicting the response of biodiversity to climate change [22]. Some of these studies ran models with more than 9 variables (Fig. 1), but the potential bias by using this HP module from R has not yet been examined. Knowing this bias is pivotal because the ranking obtained in HP is increasingly being used, for example, as a criterion for establishing priorities of conservation [13]. Because conservation resources are frequently scarce, the prioritisation assessments used to allocate conservation efforts should be highly precise [23].

In this paper, we show that this HP module produce a considerable inconsistency for analyses with more than 9 independent variables This bias is here quantified and modelled to aid researchers to make a proper assessment of the results obtained with implications in environmental management and conservation. Firstly, by means of numerical simulations we examine and quantify the robustness of the HP modules for running in R. The same set of variables in different order should produce the same results (i.e. the same ranking). Particularly, we analyze the potential variation in results obtained in relation to the order the variables are entered into the analysis. We then modelled the probability of a variable changing its position in the predictor ranking obtained in HP. Thirdly, we address these issues in two real examples worked on factors affecting the abundance and habitat selection of two threatened species. Finally, we give some recommendations to improve the analysis with this HP statistical package.

## Results

### Numerical simulations

For models with 9 or fewer variables, the nominal ranking of importance of the explanatory variables did not change with different entering order in the models (Table S1; see [18]). However, for models consisting of more than 9 variables, there was an effect of the entering order of the variables on the ranking of importance. In other words, a same variable within the same set of 10–12 variables but entered in different order changed their ranking of relative importance, based in independent explanatory power of the response variable. For models with ten variables, this nominal ranking changed, on the average, in the 90.5% of the times (SE = 0.87, range = 89–93, n = 4). For models with eleven variables the order of importance of the predictors changed, on average, in the 96.5% of the times (SE = 2.53, range = 89–100) and in the 100% (SE = 0) with twelve variables. This result changed little when the change was considered only for the five variables with most independent explanatory power (77.5%±5.86, 90.5%±5.04, 91.0%±3.76 for models with ten, eleven and twelve variables respectively).

Predictors declared as the most important by their independent explanatory power appeared in different ranking of importance in models with different orders of the same set of variables (Table 1). One predictor ranked first according to its independent variance explained (IVE hereafter) in the reference order (i.e. following alphabetic order) appeared second (6.2% of the times) and third (1.2%) with other variable orders in analysis of HP for models from ten to twelve independent variables. Predictors ranked second (in reference order) appeared in other positions in the 23.2% of the times after permuting the order of the same set of variables (see Table 1 for other ranking positions).

**Table 1:** Percentage of times that a variable changes its position within the ranking.

	Position with other variable orders											
Ranking order	1°	2°	3°	4°	5°	6°	7°	8°	9°	10°	11°	12°
	Data set-1 to Data set-4											
First	**92.5**	6.2	1.2									
Second	6.0	**76.8**	7.8	4.3	1.8	3.3	0.1					
Third	1.7	8.7	**55.7**	25.9	6.0	1.1	0.6	0.1		0.2	0.1	0.3
Fourth		3.8	24.7	**40.7**	22.9	4.8	1.8	0.8		0.2	0.3	0.8
Fifth		1.2	5.7	14.2	**42.3**	24.5	7.9	2.3	0.4	0.6	1.4	0.3
	Lesser Kestrel Data set											
AUTOCOV4	**100**											
FARMLAND		**95**	2				1	2				
DROOST		2	**70**	21	5			2				
FOREST			21	**49**	29						1	
DCOLONY10		3	9	30	**52**	4	1	1				
	Egyptian Vulture Data set											
ELEVATION	**80**	16	3									
SHRUB	21	**76**	3									
PATCH		6	**94**									
ROAD				**87**	10	1	1	1				
LENGTH				7	**47**	19	10	13	2	1		

Ranking order: Position by amount of independent explanatory power and analysed in the reference order (i.e. alphabetic order). Only the first five variables are shown.

Data set-1 to 4: numerical simulations, N = 1,200.

Lesser Kestrel Data set: N = 100, only eleven variables.

Egyptian Vulture Data set: N = 100.

The IVE varied in all the explanatory variables of all the four simulated data sets after changing the orders of the same set of variables (Table S2). For example, the IVE of the first-ranked variables changed by as little as 8.15 units (i.e., from 8.67 to 16.82) in Dataset-1 to as much as 12.4 units (i.e., from 17.97 to 30.37) in Data set-4 (see Tabla S1 for other ranking variables).

Throughout all the analysis the results were exactly the same for the different updates of the “hier.part” packages to run HP (version 1.0 and updates) [6].

### Modelling the bias

**Figure 2:**
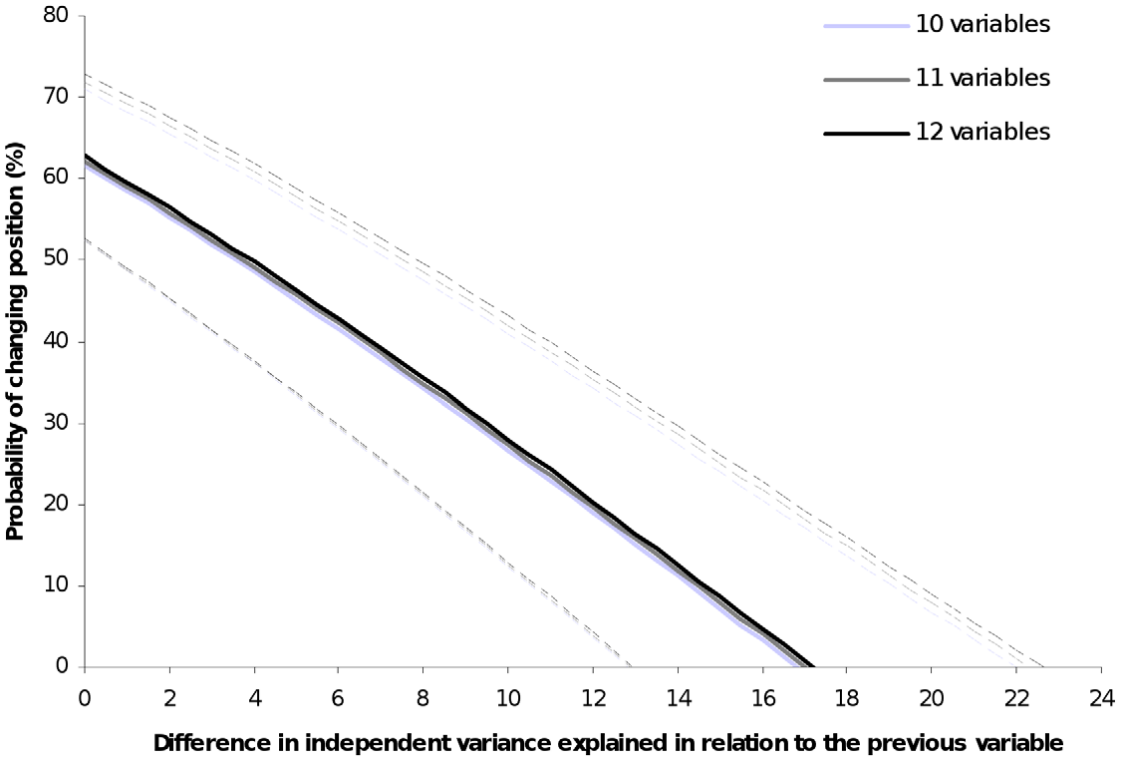
Probability of a variable changing ranking position obtained from the averaged general linear mixed model. Solid lines: Effect of the difference in independent variance explained (IVE) between a particular variable and the previous one in the ranking (established for explaining the response variable) on the probability of changing the position for data sets formed by 10, 11 and 12 explanatory variables in analysis of hierarchical partitioning. Note that no change is expected when the difference in explained variance between a variable and the previous one in the ranking is >17.1. Dotted lines: upper and lower limits according to the standard error of the averaged mixed-model coefficients.

The best models explaining the probability of a variable changing the position were those including the difference in IVE between a particular variable and the previous one in the ranking (Table 2). The lesser is this difference, the more likely is the change of position. According to our model, a decrease of one unit in the difference of IVE between a particular variable and the previous one in the ranking produces an increase of 3.7% in the probability of changing position of that variable (Fig. 2). The number of variables considered when performing HP (i.e. 10, 11 or 12) also influenced, but considerably less, the probability of changing position (Table 2). For an given amount of difference in IVE between two neighbouring variables in the ranking, the probability of changing position with 10 variables increased by 0.7% when adding one more variable (i.e. 11 variables) and by 1.3% when adding two (i.e. 12 variables; Fig. 2).

**Table 2:** Ranking of all the models explaining probability of a variable changing its position (%).

Models	AIC	AICc	ΔAICc	ω_m_	Ranking
**DIFPREVIOUS**	**94.85**	**95.14**	**0**	**0.84**	**1**
**DIFPREVIOUS + VARIABLES**	**98.77**	**99.21**	**4.07**	**0.11**	**2**
DIFPREVIOUS+DIFALL	100.5	100.94	5.80	0.05	3
DIFPREVIOUS+VARIABLES+DIFALL	106.3	106.92	11.78	0.00	4
DIFALL	112.8	113.09	17.95	0.00	5
VARIABLES+DIFALL	119.3	119.74	24.60	0.00	6
Null model	129.5	129.67	34.53	0.00	7
VARIABLES	133.4	133.69	38.55	0.00	8

AICc: Value used to rank the models.

ω_m_: Akaike weight of each model.

The best models (Σω_m_ = 0.95) are shown in bold.

### Real examples worked

**Abundance of a threatened falcon in an agricultural landscape.** In Table 3 is shown the independent (i.e., IVE), joint and total variance explained for each of the predictors of Lesser kestrel abundance in the reference order (alphabetic). The five variables that most independent variance explained in the abundance of Lesser kestrel were AUTOCOV4 (IVE = 10.63%), FARMLAND (5.98), DROOST (4.82), FOREST (4.59) and DCOLONY10 (3.71). The position of these variables in the ranking (according to percentage of IVE) changed in models with other variable orders (Table 1). The exception to this was AUTOCOV4, whose first position did not change for models with other variable orders (Table 1), although it changed the amount of IVE. IVE changed in all the variables in the ranking (Table S2). The variable that most varied the IVE among models was AUTOCOV4 ranging from 18 to 53.3% (see Table S2 for other variables).

**Table 3:** Percentage of independent, joint and total explained variance for each considered variable.

Data set-1				Lesser Kestrel Data set				Egyptian Vulture Data set			
Variables	Independent	Joint	Total	Variables	Independent	Joint	Total	Variables	Independent	Joint	Total
X_A_	4.10	−3.79	0.30	AUTOCOV4	10.63	20.85	31.48	COWS	2.49	0.48	2.97
X_B_	4.35	−3.70	0.64	BUILDUP	1.75	−0.66	1.10	ELEVATION	8.94	18.51	27.45
X_C_	4.03	−3.50	0.53	DCOLONY10	3.71	7.16	10.87	HEIGHT	1.45	−1.42	0.03
X_D_	4.05	−3.93	0.12	DROOST	4.82	4.66	9.48	LENGTH	2.74	2.03	4.77
X_E_	5.91	0.59	6.50	EDGE	1.36	−1.01	0.36	LIVESTOCK	1.67	−1.67	0.01
X_F_	5.15	−0.48	4.67	EFFORT	2.37	1.65	4.02	NEIGHBOUR	2.44	4.19	6.63
X_G_	4.96	0.40	5.37	FARMLAND	5.98	12.74	18.72	PASTURE	2.74	2.25	4.99
X_H_	4.68	−1.12	3.56	FOREST	4.59	9.91	14.50	PATCH	5.34	13.02	18.36
X_I_	8.63	9.26	17.88	GRASSLAND	1.30	−0.96	0.34	ROAD	3.67	4.36	8.03
X_J_	5.78	8.36	14.14	SHANDIVER	2.68	4.76	7.44	SHEEP	0.37	−0.30	0.07
X_K_	9.84	9.73	19.58	WIRE	2.13	−0.43	1.70	SHRUB	6.32	20.36	26.68
X_L_	10.35	16.36	26.71					SLOPE	2.09	−2.07	0.02

Total: Total explained variance, correlation (in “R^2^”) of each of the variables with the response variable (for example, X_A_, X_B_, X_C_ and X_D_ have a correlation coefficient with the response variable Y, r<0.10, i.e. each of them explain <1% of the variance of the response variable, r^2^<1%).

Joint: Negative joint variance indicates that the other variables act as suppressors of the particular variable.

Variables: The variable order shown in the tables is the order used (i.e. alphabetic order) for this particular analysis of hierarchical partitioning.

**Habitat selection by an endangered vulture.** In Table 3 is shown the independent, joint and total variance explained for each of the predictors of habitat selection by Egyptian vulture in the reference order (alphabetic). The five variables that most variance explained in habitat selection by Egyptian vulture were ELEVATION (IVE = 8.94%), SHRUB (6.32), PATCH (5.34), ROAD (3.67) and LENGTH (2.74). The position of these variables in the ranking (according to percentage of IVE) changed in models with other variable orders (Table 1). IVE also changed in all the five variables in the ranking, after permuting the order of the same set of variables. The change in IVE for each variable is given in Table S2. The variable that most changed the IVE among models was ELEVATION ranging from 11.20 to 26.43% (see Table S2 for other variables).

## Discussion

In the analyses explored here, the same set of variables in different order should produce the same results (i.e. the same ranking obtained in HP). However, for models with more than 9 variables we found that this was not true for more than 90% of the times. Specifically, a particular variable may be declared in a model as the most important predictor of the response variable but to be second or third in importance for models with the same predictors but differently ordered in around the 7.4% of the times (as extreme cases a predictor second or third in importance may be twelfth; Table 1). In variables related to both Lesser kestrel abundance and Egyptian vulture habitat selection, the results were in general the same as those of the simulated data sets, with ranking of predictors frequently changing. The only exception to this was AUTOCOV4 in the Lesser kestrel example. This variable did not change their position in the ranking with models having other variable orders (Table 1). However, this predictor considerably varied the amount of IVE among models (18–53%; Table S2). That first variable in the ranking did not change their top position in the Lesser kestrel example may be because the difference in the amount of IVE between first position and the following variable/s in the ranking (first: 10.63 *vs* second: 5.98) was larger than that of both the simulated examples (e.g. dataset-1: 10.35 *vs* 9.84) and the Egyptian vulture example (8.94 vs 6.32; Table 1 and 3). In other words, AUTOCOV4 had a larger room to vary without changing its position in the ranking (Table 1 and 3). Indeed, DROOST, FOREST and DCOLONY10 had an IVE close each other (i.e. difference in IVE, |d|<1.11) and in consequence frequently inter-changed their positions (Table 1) as predicted by our model (i.e. 58.6, 61.5 and 59.5% probability of changing position, respectively). Thereby the ranking of importance in explaining abundance of Lesser kestrel was actually uncertain for these variables. This was also the case of some variables explaining habitat selection by the Egyptian vulture. For example, ROAD and LENGTH had a similar percentage of IVE (|d|<1) and frequently inter-changed their positions in the ranking (i.e. Table 1).

### Implications in environmental management and conservation

The authors of the HP package claims that the function produces a “minor rounding error” for analyses with more than 9 independent variables [18]. In light of the results here obtained it seems that it rather produces a considerable inconsistency both quantitative and qualitative in the results. The next step, which is out of the scope of the present study, would be to investigate why the errors occur. Whilst the cause of this unexpected variation in the HP results is by the moment unclear, what is clear is that these findings need to be taken into account in future studies as well as in some already published. For example, a 20.3% of the studies using the HP package performed it with more than 9 explanatory variables in their models (Fig. 1), which might have lead to misleading conclusions about the importance of certain variables, with implications for environmental management and conservation. In fact, one of the biggest challenges faced by conservation managers today is that of the resource allocation [23]. Conservation budgets are clearly limited to correctly address all the current conservation concerns, so there is an urgent need of efficient resource allocation [24] and prioritisation assessments [23], [25]. Accordingly, knowing the correct ranking of importance of predictors is pivotal to effectively contribute to species and ecosystems conservation. In this way, it seems that the highest frequency attained in the ranking by particular predictors for models with 10–12 variables (Table 1) would be the “correct” position in the ranking, as judged by explanatory power of the predictors (Table 3). In contrast, knowing the true amount of explained variance by predictors in models with 10–12 variables is absolutely uncertain, as it hugely varies between models with same variables but differently ordered (Table S2). Therefore explained variance is not a useful parameter to use in models with more than 9 independent variables.

### Final considerations

In conclusion, if our aim is to obtain the amount of explained variance, we suggest that the HP module of R should not be used for more than 9 explanatory variables because of the inconsistency of their results. If used for establishing ranking of importance of variables it should be applied with caution, running several times the model (we suggest at least 100 times) with different order of the set of 10–12 variables (as recommended by the authors of the HP module; [18]). However, in this case it must be considered that variables with similar independent variance explained will interchange positions frequently resulting in a high uncertainty of actual positions. For 9 or fewer explanatory variables this HP module seems to work well and it is an useful tool either by itself or in combination with multiple regression analysis (e.g. GLMs), as shown by its increasing use in ecology and conservation.

## Methods

### Numerical simulations

For the analysis we used “hier.part” package (version 1.0 and updates) [6] in the R statistical software [8]. The method of fitting the model to data was by least squares (i.e. the goodness-of-fit measures were calculated by R-squared, argument: gof = “Rsqu”, and by Log-Likelihood, argument: gof = “logLik”; see [18]).

In order to assess the potential impact of the variable order on the HP results, we built 30 data sets with 13 variables each, consisting of one response variable (Y) and twelve predictor variables (the module of hierarchical partition “hier.part” for using in R does not run for more than twelve predictor variables; [18]). The variables were generated by using the multivariate Normal distribution function “rmvnorm” in SPLUS 2000; this function generates correlated random numbers. All the 13 variables had a gaussian distribution with n = 25 and standard deviation (SD) equal to 1. Different correlation patterns for each data set were generated. For example, for Data set-1, four variables (X_A_, X_B_, X_C_ and X_D_) had a correlation coefficient with the response variable (Y) r<0.10 (i.e. each of these variables explained <1% of the variance of the response variable, r^2^<1%), for the rest of variables and data sets see Table 3 and Table S2 (online appendix). The same procedure was used for the other data sets. From the 30 data sets, we randomly selected four data sets (Data set-1 to 4) for analysis (Table S3). This random selection allows us to use then mixed models for modelling the change of position fitting the “dataset” as a random effect (see below). From each data set, formed by twelve predictor variables, we selected subsamples (vectors) of 2,3,4,5,…and so on up to twelve variables by using the function “sample” in R. Then each of these eleven vectors generated were reshuffled 100 times by using the same function “sample” (i.e. the position of same variables was randomly changed within the vector), and HP was run for each of the subsamples generated (previously converted into data frames). By using this procedure, we obtained results of HP for vectors containing the same suite of variables but in different order and so it allowed us to test the potential impact of the position of the variables on HP results. Specifically, we measured how often the nominal order of relative importance of predictors (according to the amount of independent variance explained, IVE) was changed after permuting the variable order in the models. In HP, the IVE of a variable is estimated by averaging the increase in model fit over all combinations involving that variable (see [4] for a more detailed explanation). The amount of IVE of a predictor should be exactly the same independently of the entering order in models with the same suite of variables. In order to examine potential changes among models with the same suite of predictor variables but differently ordered, we used as reference model that with predictors following alphabetic order (e.g. see Table 3).

We also measured the variability in the IVE by each variable when changed its position within each of the 100 reshuffled vectors.

### Real examples worked

**Abundance of a threatened falcon in an agricultural landscape.** Data of this example come from a previous paper [16]. In this example, we examine the spatial pattern of Lesser kestrel (*Falco naumanni*) abundance in function of a set of environmental factors at landscape level (Table S4). This species is a small falcon breeding in the Palaearctic and wintering mainly in Africa [26], [27], and is considered to be a globally threatened species listed as Vulnerable [28]. The study area (384 km^2^) was divided into 24 contiguous UTM grid 4km×4 km (16km^2^) squares, where birds were counted in up to 3 visits per square. An index of relative density (IRD, no. of Lesser kestrels in 1 km of driven transect) was calculated for each grid and each visit. Then, the averages of IRD of the visits performed per square were calculated for each grid (response variable). Environmental variables were measured within strips 250 m wide at each side of the routes for censusing kestrels and were extracted with aid of a geographic information system (GIS software, ARCGIS 8.0). Eleven independent variables were considered (Table S4). Lesser kestrel abundance was log-transformed (ln[*x*+0.5]) to reach normal distribution. The method of fitting the model to data in HP was by least squares (i.e. the goodness-of-fit measures were calculated by R-squared).

**Habitat selection by an endangered vulture.** Data of this example come from a previous paper [29]. Here we developed habitat-occupancy models for the Egyptian vulture (*Neophron percnopterus*) using a set of environmental factors describing both the nesting cliff and the surrounding landscape (Table S4). The Egyptian vulture is a territorial, cliff-nesting, migrant scavenger raptor distributed from the Mediterranean countries to India, occupying also areas in the east and south of Africa. It has been recently classified as Endangered by IUCN [30]. We selected 62 Egyptian vulture breeding territories (presences) within the study area (8500 km^2^) and 58 randomly generated points without apparent Egyptian vulture breeding pairs (pseudo-absences). Variables describing the nesting cliff and the home range (i.e. 2.5 km radius around the nest) were extracted using ARCGIS 9.0 and were validated by field observations when necessary. Twelve variables were considered (Table S4). The assumption of linearity was evaluated by plotting response variable (i.e. presence/absence) against every continuous explanatory variable in Generalized Additive Models (GAMs) [31]. Non-linear relationships were converted into piece-wise linear effects, with the best threshold being selected according to the lowest residual deviance [32]. The maximum likelihood method was used to fit the model to data in HP (i.e. argument of goodness-of-fit “logLik”).

### Modelling the bias

We defined bias as the probability of a variable changing its position. This was measured as percentage of times a particular variable changed its position within the predictor ranking obtained in HP (see above). In order to assess factors influencing probability of a variable's position change, we used five data set (i.e. Data set-1 to Data set-4 and Lesser kestrel data set) and performed generalized linear mixed models (GLMM). We modelled the probability of position change (%, dependent variable) as a function of seven explanatory variables (fixed effects) accounting for the number of variables (10–12) and for differences in amount of IVE between explanatory variables (Table 4). We used the data set (i.e. 5 levels, the 4 simulated data sets plus the Lesser kestrel data set) as a random effect. This allowed us to control for the possibility that the probability of changing position might vary due to factors related to the data set itself, whose effects are not taken into account in the fixed effects. Random effects allow us also control for the fact that variables (10–12) within same data set could be pseudo-replicates. Importantly, considering the data set as a random effect, our results can thus be extrapolated to a population of data sets from which our sample was drawn. We used *lmer* function of “lme4” package with an identity link function and a gaussian error distribution. The probability of changing position was transformed into asin(x) to reach normal distribution. First, we used the Spearman's rank correlation to explore the correlations between the variables.The highly correlated variables (|r_s_|>0.5) were included separately in the models (i.e. they were not put together in the same model). We performed all possible model permutations of the explanatory variables. Resultant models were ranked altogether using the AICc and the Akaike weight of each model (ω_m_) [33], estimated following Burnham and Anderson [34]. Akaike weight is the relative likelihood of that model being the Kullback-Leibler best model within a set of *n* models, with ω_m_>0.9 indicating a high level of support for a given model. We constructed a 95% confidence set of models by starting with the highest Akaike weight and adding the model with the next highest weight until the cumulative sum of weights exceeded 0.95 [34]. We used these final best models to obtain the averaged model which was used for inference. Additionally, we examined models with non-linear variables (second-order polynomial), which were no better (according to AIC) than those with linear variables, so only linear variables were considered.

**Table 4:** Variables used for explaining the probability of a variable changing its ranking position.

Variable	Definition
DIFNEXT	Difference in independent variance explained between a variable and the next one
DIFPREVIOUS	Difference in independent variance explained between a variable and the previous one
DIFSECOND	Difference in independent variance explained between a variable and the second-further one
DIFTHIRD	Difference in independent variance explained between a variable and the third-further one
DIFNEXTPREVIOUS	Sum of the differences in independent variance explained between a variable and the next and previous ones
DIFALL	Sum of the differences in independent variance explained between a variable and all the rest
VARIABLES	Number of variables used to perform the HP analysis

## Supporting Information

Table S1 Number of times the ranking of predictors changed relative to the reference order (i.e. that following order alphabetic for analysis) when other variable orders were analysed by hierarchical partitioning for models from two to twelve predictors. Numbers are averaged percentages (and range) of the number of times the ranking of predictors changed (n = 100 for each suite of predictors and Dataset, n = 4 Datasets)
(0.03 MB DOC)

Table S2 Mean, standard deviation and range of the percentage in independent explained variance of the five best predictors obtained after permuting 100 times the variable order for each dataset. Note that the results with 10, 11 and 12 variables for each simulated dataset (i.e. Dataset-1 to Dataset-4) are pulled together.
(0.07 MB DOC)

Table S3 Independent, joint and total variance explained (in percentage) for each of the variables taken into account in the numerical simulation from Dataset-2 to Dataset-4. Note that the total explained variance (Total column) expresses the correlation (in “R2”) of each of the variables with the response variable. Negative joint variance indicates that the other variables act as suppressors of the particular variable.
(0.05 MB DOC)

Table S4 Independent variables used for explaining the distribution of lesser kestrel abundance in a Spanish farmland and habitat selection by Egyptian vulture in a mountain area.
(0.05 MB DOC)
