# Supplementary material for: Estimating and Modelling Bias of the Hierarchical Partitioning Public-Domain Software: Implications in Environmental Management and Conservation
Source: PLoS One. 2010 Jul 21;5(7):e11698. doi: 10.1371/journal.pone.0011698 (PMC2908144; doi:10.1371/journal.pone.0011698)
Supplement: Table S1 — Number of times the ranking of predictors changed relative to the reference order (i.e. that following order alphabetic for analysis) when other variable orders were analysed by hierarchical partitioning for models from two to twelve predictors. Numbers are averaged percentages (and range) of the number of times the ranking of predictors changed (n = 100 for each suite of predictors and Dataset, n = 4 Datasets) (0.03 MB DOC) [file pone.0011698.s001.doc]

Table S1

| Number of predictors | averaged percentage (range)* | averaged percentage  (range)§ |
| --- | --- | --- |
| Two | 0 (0-0) | 0 (0-0) |
| Three | 0 (0-0) | 0 (0-0) |
| Four | 0 (0-0) | 0 (0-0) |
| Five | 0 (0-0) | 0 (0-0) |
| Six | 0 (0-0) | 0 (0-0) |
| Seven | 0 (0-0) | 0 (0-0) |
| Eight | 0 (0-0) | 0 (0-0) |
| Nine | 0 (0-0) | 0 (0-0) |
| Ten | 91.25 (89-93) | 62.75 (21-89) |
| Eleven | 96.5 (89-100) | 77.5 (24-99) |
| Twelve | 100 (100-100) | 91(82-100) |

*Considering all the predictors

§ Considering first five predictors most important by independent explanatory power
