# Supplementary material for: Estimating and Modelling Bias of the Hierarchical Partitioning Public-Domain Software: Implications in Environmental Management and Conservation
Source: PLoS One. 2010 Jul 21;5(7):e11698. doi: 10.1371/journal.pone.0011698 (PMC2908144; doi:10.1371/journal.pone.0011698)
Supplement: Table S2 — Mean, standard deviation and range of the percentage in independent explained variance of the five best predictors obtained after permuting 100 times the variable order for each dataset. Note that the results with 10, 11 and 12 variables for each simulated dataset (i.e. Dataset-1 to Dataset-4) are pulled together. (0.07 MB DOC) [file pone.0011698.s002.doc]

Table S2

|  | Mean | SD | Minimum | Maximum |
| --- | --- | --- | --- | --- |
| Dataset-1 | | | | |
| First | 10.80 | 1.98 | 8.67 | 16.82 |
| Second | 9.69 | 1.53 | 7.97 | 13.61 |
| Third | 7.37 | 1.86 | 5.38 | 12.57 |
| Fourth | 4.44 | 1.40 | 2.75 | 8.26 |
| Fifth | 3.82 | 0.89 | 2.65 | 6.21 |
| Dataset-2 | | | | |
| First | 13.83 | 1.94 | 11.97 | 20.36 |
| Second | 6.13 | 1.32 | 4.90 | 10.82 |
| Third | 5.28 | 1.17 | 4.00 | 9.10 |
| Fourth | 3.91 | 0.95 | 2.73 | 7.04 |
| Fifth | 3.09 | 1.06 | 1.87 | 6.80 |
| Dataset-3 | | | | |
| First | 14.35 | 2.66 | 11.58 | 21.07 |
| Second | 7.58 | 1.48 | 5.14 | 11.25 |
| Third | 7.63 | 2.23 | 4.67 | 14.15 |
| Fourth | 6.94 | 1.68 | 4.46 | 11.08 |
| Fifth | 5.41 | 1.75 | 2.85 | 10.28 |
| Dataset-4 | | | | |
| First | 21.43 | 3.55 | 17.97 | 30.37 |
| Second | 7.92 | 2.33 | 5.55 | 14.48 |
| Third | 3.81 | 1.92 | 2.04 | 9.30 |
| Fourth | 3.86 | 2.05 | 2.19 | 10.03 |
| Fifth | 2.86 | 1.77 | 1.32 | 8.25 |
| Lesser Kestrel Dataset | | | | |
| AUTOCOV4 | 27.62 | 4.18 | 18.00 | 53.30 |
| FARMLAND | 13.75 | 1.63 | 7.15 | 16.50 |
| DROOST | 10.93 | 1.16 | 6.90 | 14.34 |
| FOREST | 10.06 | 0.94 | 4.92 | 11.88 |
| DCOLONY10 | 9.48 | 1.05 | 5.66 | 11.65 |
| Egyptian Vulture Dataset | | | | |
| ELEVATION | 19.01 | 3.46 | 11.20 | 26.43 |
| SHRUB | 17.13 | 2.04 | 11.85 | 21.09 |
| PATCH | 12.61 | 1.17 | 9.23 | 14.55 |
| ROAD | 9.13 | 1.06 | 6.40 | 11.98 |
| LENGHT | 7.08 | 0.73 | 4.67 | 9.68 |
