# Supplementary material for: Estimating and Modelling Bias of the Hierarchical Partitioning Public-Domain Software: Implications in Environmental Management and Conservation
Source: PLoS One. 2010 Jul 21;5(7):e11698. doi: 10.1371/journal.pone.0011698 (PMC2908144; doi:10.1371/journal.pone.0011698)
Supplement: Table S3 — Independent, joint and total variance explained (in percentage) for each of the variables taken into account in the numerical simulation from Dataset-2 to Dataset-4. Note that the total explained variance (Total column) expresses the correlation (in “R2”) of each of the variables with the response variable. Negative joint variance indicates that the other variables act as suppressors of the particular variable. (0.05 MB DOC) [file pone.0011698.s003.doc]

| Dataset-2 | | | |  | Dataset-3 | | | |  | Dataset-4 | | | |
| --- | --- | --- | --- | --- | --- | --- | --- | --- | --- | --- | --- | --- | --- |
| Variables | Independent | Joint | Total |  | Variables | Independent | Joint | Total |  | Variables | Independent | Joint | Total |
| X_A_ | 5.24 | 9.33 | 14.56 |  | X_A_ | 5.63 | 8.00 | 13.62 |  | X_A_ | 1.44 | -1.44 | 0.00 |
| X_B_ | 3.21 | -1.03 | 2.17 |  | X_B_ | 2.14 | -1.36 | 0.78 |  | X_B_ | 1.51 | -1.02 | 0.49 |
| X_C_ | 10.85 | 15.07 | 25.92 |  | X_C_ | 10.15 | 19.17 | 29.32 |  | X_C_ | 2.16 | 1.62 | 3.78 |
| X_D_ | 3.90 | 1.75 | 5.65 |  | X_D_ | 3.02 | -0.23 | 2.79 |  | X_D_ | 1.70 | 0.43 | 2.13 |
| X_E_ | 9.92 | 5.93 | 15.85 |  | X_E_ | 5.42 | 4.33 | 9.76 |  | X_E_ | 15.28 | 37.91 | 53.19 |
| X_F_ | 3.26 | -1.58 | 1.68 |  | X_F_ | 2.13 | -1.99 | 0.13 |  | X_F_ | 1.49 | -1.01 | 0.47 |
| X_G_ | 3.40 | -3.23 | 0.17 |  | X_G_ | 4.29 | 0.17 | 4.45 |  | X_G_ | 2.74 | 1.94 | 4.68 |
| X_H_ | 3.24 | -1.98 | 1.26 |  | X_H_ | 4.37 | 3.11 | 7.48 |  | X_H_ | 1.42 | -1.19 | 0.22 |
| X_I_ | 9.62 | 14.56 | 24.18 |  | X_I_ | 3.33 | -0.97 | 2.36 |  | X_I_ | 6.09 | 12.40 | 18.48 |
| X_J_ | 4.01 | -3.01 | 1.00 |  | X_J_ | 4.08 | 7.59 | 11.67 |  | X_J_ | 2.61 | 4.30 | 6.91 |
| X_K_ | 2.86 | -1.15 | 1.71 |  | X_K_ | 6.11 | 11.51 | 17.62 |  | X_K_ | 1.90 | 0.30 | 2.20 |
| X_L_ | 3.62 | 2.21 | 5.83 |  | X_L_ | 3.67 | -3.66 | 0.01 |  | X_L_ | 3.18 | 4.26 | 7.44 |

Table S3
