# Supplementary material for: Estimating and Modelling Bias of the Hierarchical Partitioning Public-Domain Software: Implications in Environmental Management and Conservation
Source: PLoS One. 2010 Jul 21;5(7):e11698. doi: 10.1371/journal.pone.0011698 (PMC2908144; doi:10.1371/journal.pone.0011698)
Supplement: Table S4 — Independent variables used for explaining the distribution of lesser kestrel abundance in a Spanish farmland and habitat selection by Egyptian vulture in a mountain area. (0.05 MB DOC) [file pone.0011698.s004.doc]

| ***Variable*** | ***Definition*** |
| --- | --- |
| **Lesser Kestrel Dataset** |  |
| FARMLAND | Surface in % of farmland |
| GRASSLAND | Surface in % of grassland |
| FOREST | Surface in % of forest |
| BUILD-UP | Surface in % of build-up areas |
| EDGE | Total edge length in km of all habitat patches per km^2^ |
| SHANDIVER | Habitat diversity using Shannon’s diversity index |
| WIRE | Total length in km of electrical wires |
| DCOLONY10 | Distance in km to the nearest colony with more than 10 breeding pairs from the centre of each grid square |
| DROOST | Distance in km to the nearest roost from the centre of each grid square |
| AUTOCV4 | Average of kestrel IRD in the 8 nearest neighbour squares; it allows to explain for spatial autocorrelation |
| EFFORT | No. of visits in each square to count birds |
| **Egyptian Vulture Dataset** |  |
| HEIGHT | Height of the nesting cliff at the level of the nest (m) |
| LENGHT | Longitude of the nesting cliff (m) |
| ELEVATION | Altitude of the cliff (m.a.s.l.) |
| NEIGHBOUR | Linear distance (m) between the nest and the nearest Egyptian Vulture nest occupied that year |
| SLOPE | Mean slope within a radius of 2.5 km around the nest (in degrees) |
| SHRUB | Percentage of shrubland within a radius of 2.5 km |
| PASTURE | Percentage of surface covered by pastures within a radius of 2.5 km |
| PATCH | Number of landscape patches of any type within a radius of 2.5km |
| LIVESTOCK | Number of livestock units within a radius of 2.5km that year. 1 cow =5 livestock units; 1 sheep or goat=1 livestock unit |
| COWS | Number of livestock units of cow within a radius of 2.5km that year |
| SHEEP | Number of livestock units of sheep and goat within a radius of 2.5 km that year |
| ROAD | Density of paved road (m) within a radius of 2.5 km |

Table S4
